# Supplementary material for: Causal Relationship between Aging and Anorexia Nervosa: A White-Matter-Microstructure-Mediated Mendelian Randomization Analysis
Source: Biomedicines. 2024 Aug 16;12(8):1874. doi: 10.3390/biomedicines12081874 (PMC11351342; doi:10.3390/biomedicines12081874)
Supplement: Supplementary file 1 [file biomedicines-12-01874-s001.zip › Supplementary Figure/Figure S4/Singe SNP plot of MR analysis for TL to ubm-b-2028.pdf]

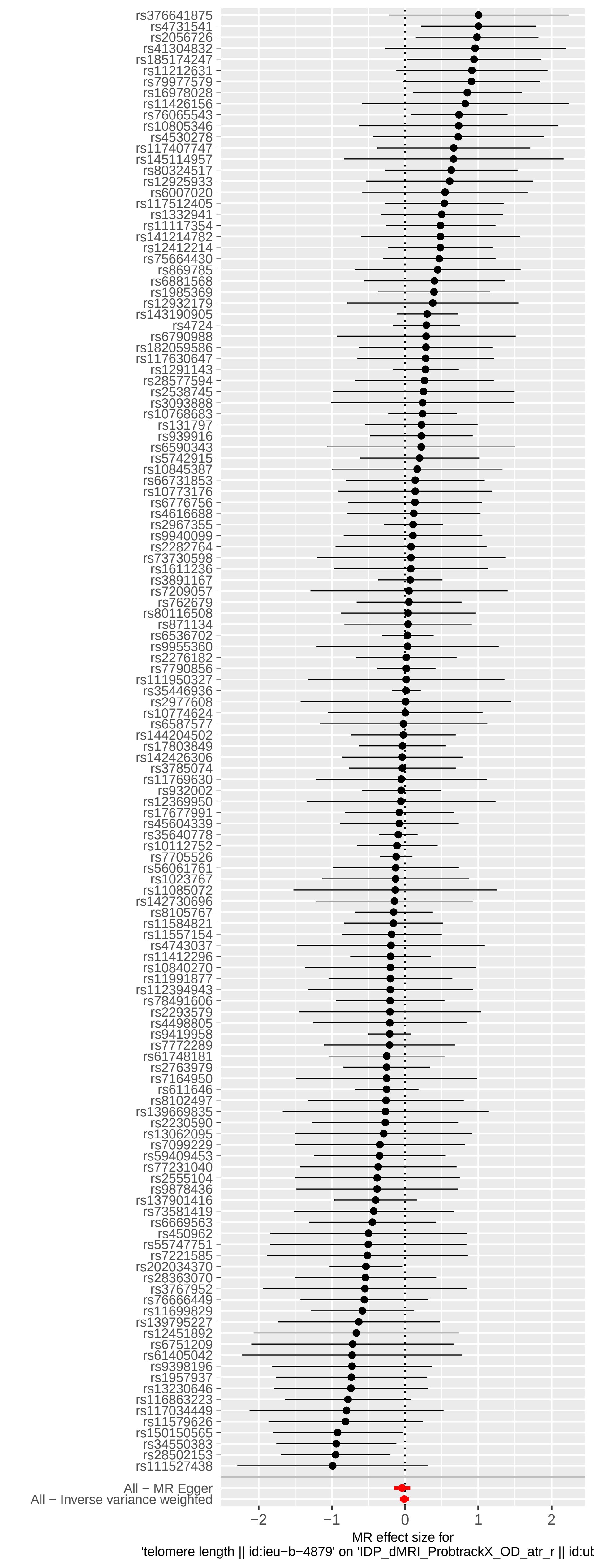
rs376641875
rs4731541
rs2056726
rs41304832
rs185174247
rs11212631
rs79977579
rs16978028
rs11426156
rs76065543
rs10805346
rs4530278
rs117407747
rs145114957
rs80324517
rs12925933
rs6007020
rs117512405
rs1332941
rs11117354
rs141214782
rs12412214
rs75664430
rs869785
rs6881568
rs1985369
rs12932179
rs143190905
rs4724
rs6790988
rs182059586
rs117630647
rs1291143
rs28577594
rs2538745
rs3093888
rs10768683
rs131797
rs939916
rs6590343
rs5742915
rs10845387
rs66731853
rs10773176
rs6776756
rs4616688
rs2967355
rs9940099
rs2282764
rs73730598
rs1611236
rs3891167
rs7209057
rs762679
rs80116508
rs871134
rs6536702
rs9955360
rs2276182
rs7790856
rs111950327
rs35446936
rs2977608
rs10774624
rs6587577
rs144204502
rs17803849
rs142426306
rs3785074
rs11769630
rs932002
rs12369950
rs17677991
rs45604339
rs35640778
rs10112752
rs7705526
rs56061761
rs1023767
rs11085072
rs142730696
rs8105767
rs11584821
rs11557154
rs4743037
rs11412296
rs10840270
rs11991877
rs112394943
rs78491606
rs2293579
rs4498805
rs9419958
rs7772289
rs61748181
rs2763979
rs7164950
rs611646
rs8102497
rs139669835
rs2230590
rs13062095
rs7099229
rs59409453
rs77231040
rs2555104
rs9878436
rs137901416
rs73581419
rs6669563
rs450962
rs55747751
rs7221585
rs202034370
rs28363070
rs3767952
rs76666449
rs11699829
rs139795227
rs12451892
rs6751209
rs61405042
rs9398196
rs1957937
rs13230646
rs116863223
rs117034449
rs11579626
rs150150565
rs34550383
rs28502153
rs111527438
All − MR Egger
All − Inverse variance weighted
−2
−1
0
1
2
MR effect size for
'telomere length || id:ieu-b-4879' on 'IDP_dMRI_ProbtrackX_OD_atr_r || id:ub
